# Exaggerated object affordance and absent automatic inhibition in alien hand syndrome

**DOI:** 10.1016/j.cortex.2013.01.004

**Published:** 2013-09

**Authors:** Jennifer McBride, Petroc Sumner, Stephen R. Jackson, Nin Bajaj, Masud Husain

**Affiliations:** a Institute of Cognitive Neuroscience, University College London, Alexandra House, London, UK; b Institute of Neurology, University College London, Alexandra House, London, UK; c School of Psychology, Cardiff University, Tower Building, Park Place, Cardiff, UK; d School of Psychology, University of Nottingham, Nottingham, UK; e Department of Clinical Neurology, Nottingham University Hospitals NHS Trust, Queen's Medical Centre, Nottingham, UK; f Nuffield Department of Clinical Neurosciences, University of Oxford, UK; g Department of Experimental Psychology, University of Oxford, UK

**Keywords:** Alien limb, Object affordance, Automatic inhibition, Masked priming

## Abstract

Patients with *alien hand syndrome* (AHS) experience making apparently deliberate and purposeful movements with their hand against their will. However, the mechanisms contributing to these involuntary actions remain poorly understood. Here, we describe two experimental investigations in a patient with corticobasal syndrome (CBS) with alien hand behaviour in her right hand. First, we show that responses with the alien hand are made significantly more quickly to images of objects which afford an action with *that* hand compared to objects which afford an action with the unaffected hand. This finding suggests that involuntary grasping behaviours in AHS might be due to exaggerated, automatic motor activation evoked by objects which afford actions with that limb. Second, using a backwards masked priming task, we found normal automatic inhibition of primed responses in the patient's unaffected hand, but importantly there was no evidence of such suppression in the alien limb. Taken together, these findings suggest that grasping behaviours in AHS may result from exaggerated object affordance effects, which might potentially arise from disrupted inhibition of automatically evoked responses.

## 1 Introduction

Although most healthy adults feel that they have a great deal of control over their actions, some neurological patients do not. Patients with *alien hand syndrome* (AHS) may involuntarily grasp objects placed within their reach, experiencing difficulty releasing objects once grasped (see e.g., Biran and Chatterjee, 2004; Della Sala et al., 1991). Despite the fact that such individuals make seemingly deliberate and purposeful movements with their “alien” hand, there is clear disparity between actions performed by the alien limb and the intentions of patients, who subjectively report that the hand is not under their control. Instead, they report that the alien limb behaves as though it has a mind of its own or is being controlled by an external agent (e.g., Assal et al., 2007; Biran and Chatterjee, 2004; Fitzgerald et al., 2007). Although these remarkable grasping behaviours in AHS are now well-documented, we understand very little about the mechanisms that might underlie such behaviour.

AHS is a relatively rare syndrome (for a review, see Fisher, 2000), so detailed investigation has been correspondingly sparse. Some of the most detailed experimental work comes from Riddoch and her colleagues (e.g., Humphreys and Riddoch, 2000; Riddoch et al., 1998). They instructed a patient with bilateral AHS to reach out and grasp a cup with a hand. The patient was able to do this correctly as long as the cup's handle was on the same side as the hand they were instructed to use to grasp the cup. However, if the handle was on the opposite side, “interference” errors were generated with the patient reaching with whichever hand matched the side the cup's handle was on. For example, if instructed to grasp a cup with the right hand when the cup's handle was to the left, the patient would often erroneously grasp the cup with the left hand. These effects are unlikely to be perceptual or attentional because fewer interference errors were made when the task was to point rather than to grasp, or when the patient responded to LEDs instead of to cups. These findings suggest that, for this patient, simple observation of a graspable object might be sufficient to elicit the associated motor plan for interacting with that object, even when the plan conflicts with current goals (see also Blakemore et al., 2002).

Indeed, such involuntary grasping behaviour in AHS may be related to the longstanding view that, even in healthy adults, viewing visual objects can automatically prime actions in the observer. AHS might represent an exaggerated form of such automatic priming. Gibson (1979) described “affordances” as properties of objects in the environment which prime an observer to act. For example, seeing a teapot with the handle to the right might automatically prime the observer to reach out with the right hand to grasp the handle. Object affordance effects such as these have been extensively studied in healthy adults using stimulus-response compatibility paradigms (e.g., Cho and Proctor, 2010; Derbyshire et al., 2006; Iani et al., 2011; McBride et al., 2012b; Pellicano et al., 2010; Phillips and Ward, 2002; Tucker and Ellis, 1998, 2001). For example, Tucker and Ellis (1998) presented pictures of objects which healthy observers classified as upright or inverted as quickly and accurately as possible using a manual button press. Crucially, the objects could be presented so that they maximally afforded a response with either the left or the right hand. Although this left/right orientation was irrelevant to the participants' task, responses were significantly faster and more accurate when participants responded with a hand that was congruent with the (task-irrelevant) response afforded by the object.

These findings, and the many others like them (e.g., Cho and Proctor, 2010; Derbyshire et al., 2006; Iani et al., 2011; McBride et al., 2012b; Pellicano et al., 2010; Phillips and Ward, 2002; Tucker and Ellis, 1998, 2001), suggest that through experience observers associate objects with particular actions, and that these actions can be (partially) evoked by perceptual processing of the object even when they are irrelevant to the observer's task. Of course, in healthy people, objects do not always elicit actions towards them; that would make people entirely stimulus-bound. Hence there is a need to suppress such automatically evoked affordances. Indeed in healthy observers, there is now compelling evidence that responses automatically primed by the environment can also be automatically suppressed (for reviews see Eimer and Schlaghecken, 2003; McBride et al., 2012a; Sumner, 2007).

Using a backwards masked priming paradigm, Eimer and Schlaghecken (1998) showed that participants' responses to targets were typically speeded if targets were preceded by a compatible prime (a prime associated with the same response as the target) compared to when targets followed an incompatible prime (a prime associated with the opposite response to the target). Thus, a target directing a left hand response is faster if preceded by a (backward-masked) left prime relative to a right prime. However, when the interval between masked-prime and target is extended beyond ∼150 msec this usual positive compatibility effect (PCE) actually *reverses* to produce a negative compatibility effect (NCE; Eimer and Schlaghecken, 1998). Now, a target directing a left hand response is actually slower if it is preceded by a (backward-masked) left prime relative to a right prime.

As long as appropriate stimuli are used (see Schlaghecken et al., 2007; Lleras and Enns, 2004; Sumner, 2008), this NCE can be interpreted as reflecting automatic suppression of the primed response (see e.g., Eimer and Schlaghecken, 2003; Jáskowski, 2007, 2008, 2009; Sumner, 2007). According to these sensorimotor accounts of the NCE, initial motor activation evoked by the prime is subsequently suppressed when the prime is removed or a novel stimulus (the mask) is added to the scene (e.g., Boy et al., 2008; Jáskowski, 2007, 2008, 2009). This suppression means that it takes longer to initiate the suppressed response relative to a response which has not been inhibited, thereby producing the NCE.

Sumner and Husain (2008) suggested that such automatic suppression of automatically evoked responses might be crucial for goal-directed behaviour because it frees an organism from stimulus-bound responses, and provides a level playing field for alternative actions to occur according to the current goals of an animal. Consistent with this proposal, Vainio and colleagues have reported that automatic inhibition is not restricted to masked-prime paradigms, but also occurs when responses are afforded by graspable stimuli (e.g., Vainio, 2009; Vainio et al., 2011; Vainio and Mustonen, 2011).

Such considerations naturally raise the possibility of grasping behaviour in AHS arising from disruption of automatic inhibitory mechanisms which, in healthy observers, halt inappropriate activation of responses afforded by the environment (see also Blakemore et al., 2002; Giovannetti et al., 2005). At present, however, there is very little direct evidence to support this hypothesis, although there are some suggestive pieces of evidence. In healthy adults, the supplementary motor area (SMA) in the medial frontal lobes is associated both with simply viewing graspable objects without reaching for them (e.g., Grèzes and Decety, 2002) as well as with successful automatic inhibition of primed responses indexed by the NCE (e.g., Boy et al., 2010a, 2011; Sumner et al., 2007). Intriguingly, AHS has long been associated with damage to these same medial frontal regions (e.g., Bakheit et al., 2013; Marchetti and Della Sala, 1998). AHS is increasingly recognised in corticobasal syndrome (CBS, to distinguish it from the pathologic entity, corticobasal degeneration, CBD; see Boeve et al., 2003). CBS is a rare (annual incidence rates have been estimated at around .02 per 100,000 individuals; Winter et al., 2010), slowly progressive, neurodegenerative condition which affects cortical regions as well as the basal ganglia (e.g., Fitzgerald et al., 2007; Murray et al., 2007; Riddoch et al., 1998; Tiwari and Amar, 2008). Interestingly, CBS is also associated with metabolic impairment in the SMA (e.g., Garraux et al., 2000).

To the best of our knowledge, patients with AHS have not previously been tested on object affordance “compatibility” tasks, or paradigms designed to investigate automatic inhibition of primed actions (e.g., masked priming). We met with four patients with CBS (see Table 1 for a summary of patients' details), but unfortunately the motor symptoms experienced by three of these patients were so severe that they were not able to complete basic motor tasks. However, one patient, Patient SA, was able to make speeded manual responses with either hand according to stimuli presented. Patient SA had AHS which affected her right hand (involuntary grasping movements to objects placed within her reach), and no evidence of alien behaviour in her left hand (see Table 1).

Here we report results from two experiments conducted with Patient SA. Experiment 1 was designed to investigate whether object affordance effects were stronger in the alien hand relative to the unaffected hand. Our second study compared automatic inhibition of action in the two hands. If grasping behaviour in AHS arises because of disruption of normal automatic suppression of afforded responses, one might predict that (i) object affordance effects are exaggerated in the alien hand compared to the non-alien hand (and relative to healthy controls); and (ii) automatic inhibition of automatically evoked responses is reduced in the alien limb.

## 2 Case report

Patient SA was a 72-year-old, right-handed woman who first reported noticing her symptoms 3 years previously when she had a fall. At that time, it was observed that her speech had a telegraphic quality. She developed progressive difficulty speaking and writing, swallowing, and controlling her right hand. She began to use her right arm less frequently. Although she could voluntarily move it if necessary, there was a lack of spontaneous use. Soon, she began to experience difficulty chopping vegetables using the right hand. She encountered problems with her right hand grip, but at that time had no difficulty letting objects go. Prior to testing, she noted that her walking had slowed. She began to experience difficulties standing from a seated position. There was no family history of neurodegenerative disease.

On examination, she had a profound expressive aphasia and impaired articulation. However, she was able to comprehend 3-stage commands well. Visual fields were full to confrontation. There was no evidence of visual or tactile extinction. Eye movements were full, but she was slow to initiate saccades, particularly towards the left compared to the right and there was some evidence of gaze impersistence. Such oculomotor deficits are not uncommon in CBS patients. There was no facial weakness and palatal movements were normal. There was no pout reflex.

There was rigidity of the right arm and poor fine finger movements, but good strength throughout. The right hand showed evidence of mild alien hand behaviour, with involuntary grasping of any object that was brought close to it. The patient was adamant that she was not willing the hand to do this, and she could not stop this behaviour even when she made an effort to do so. There was no evidence of alien hand behaviour in the left hand.

Examination did not reveal any dystonia or limb apraxia, above and beyond the problems associated with fine control of the right hand movements. There was no amorphosynthesis in the left hand. When she walked, there was reduced arm swing, more prominently on the right than on the left, but she had a good stride length and postural reflexes were intact. There was no evidence of some of the other behaviours which are common in AHS: no levitation of either arm, no mirror movements, and no intermanual conflict between the hands. Overall, the clinical presentation was considered to be consistent with CBS.

**Fig. 1:**
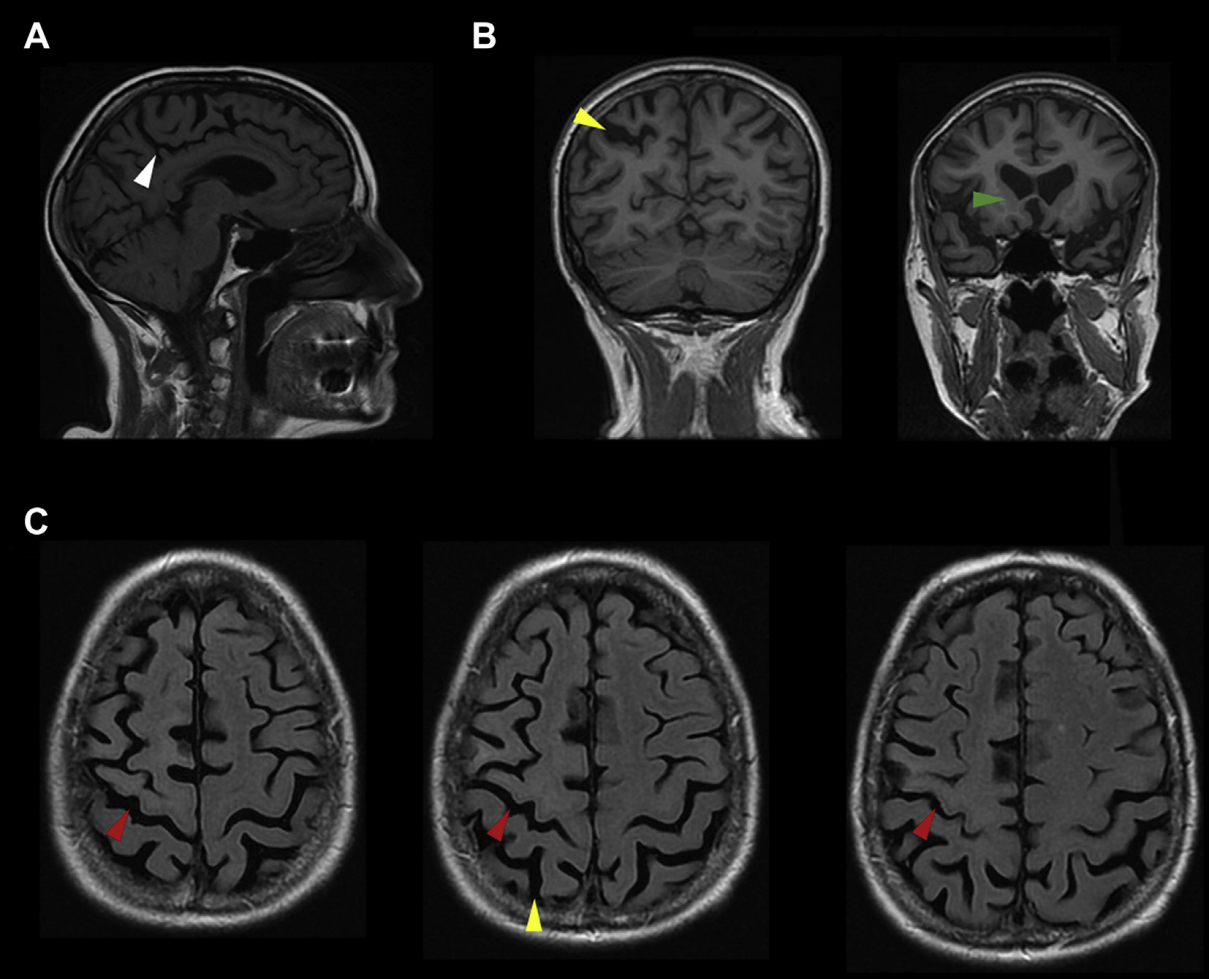
**MRI brain scans of Patient SA**. (A) Sagittal image demonstrating loss of volume of the left medial frontal and parietal cortex with a pathologically widened cingulate sulcus. (B) Coronal images showing loss of cortical volume particularly of the superior parietal lobe adjacent to a widened intraparietal sulcus, most prominently on the left, together with reduction in caudate head volume bilaterally. (C) Axial images demonstrating widened sulci over superior parietal and frontal regions, including the left central sulcus. White arrowhead = cingulate sulcus; yellow arrowhead = left intraparietal sulcus; red arrowhead = left central sulcus; green arrowhead = caudate head.

Magnetic resonance imaging (MRI; Fig. 1) demonstrated cortical atrophy, slightly more prominent over parietal than frontal regions and in the left hemisphere compared to the right. In addition, there was reduction in volume of the caudate head bilaterally. These findings would be consistent with the clinical diagnosis of CBS. Selected images in Fig. 1 demonstrate loss of volume of the left medial frontal and parietal cortex with a pathologically widened cingulate sulcus (white arrowhead); loss of cortical volume adjacent to a widened intraparietal sulcus particularly involving the superior parietal lobe, most prominently on the left (yellow arrowhead); widened sulci over superior parietal and frontal regions, including the left central sulcus (red arrowhead); and reduction in caudate head volume bilaterally (left side marked with green arrowhead).

SA completed the two different experiments on two different days, approximately 4 weeks apart. The affordance task was performed first. This study was approved by the local human subjects ethics committee and the patient gave written informed consent prior to testing.

## 3 Experiment 1|Object affordance task

Stimuli, task, response measurement and analysis follow closely from those reported in McBride et al. (2012b) which reported data from young healthy individuals.

### 3.1 Stimuli and task

**Fig. 2:**
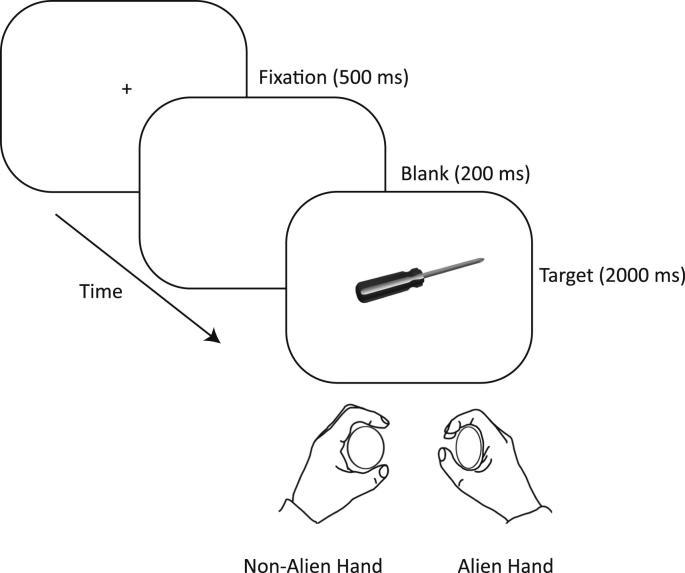
**Sequence of events in a typical affordance experiment trial**. Patient SA was instructed to make a squeeze response with the left hand if the object presented belonged in a kitchen, and to make a squeeze response with the right hand if the object belonged in a toolbox (the latter is depicted here). Each object could be presented in two possible orientations, so that it afforded an action with the left or right hand (all trial types were equiprobable). Object orientation was irrelevant to the task.

Each trial began with presentation of a black fixation cross on a white background on a CRT monitor (see Fig. 2). This cross subtended 1 degree × 1 degree of visual angle, and was presented in the centre of the screen for 1500 msec. Following a blank interval (200 msec), an image of a target object was presented at screen centre for 2000 msec. Stimuli were pictures of ten household objects taken from the Object Databank (courtesy of Michael J. Tarr, Brown University, http://www.tarrlab.org/) and Verfaille and Bousten's 3D object database (see Verfaillie and Boutsen, 1995; Boutsen et al., 1998). Objects were matched for orientation. Five objects belonged in a kitchen (fork, frying pan, knife, saucepan, spoon), and five in a toolbox (chisel, pliers, saw, screwdriver, spanner). Images subtended 10.6–17.3 degrees of visual angle horizontally, and 2.8–5.3 degrees of visual angle vertically. Objects were oriented with their handles affording an action with the left or right hand.

The participant was instructed to respond by making a short, sharp squeeze of a grip force measuring device (details below) with the left hand for kitchen objects, and with the right hand for toolbox objects. Therefore, depending on the orientation of the object presented, the object could afford an action that was either “congruent” or “incongruent” with the required response. The next trial began following a blank interval (1000 msec).

Before the experiment began, the participant practiced making responses while observing the output from the pressure transducers on a computer screen. Following a short practice block (12 trials) Patient SA completed two sessions on the same day, each containing 4 blocks of 64 trials each, totalling 512 trials after practice. There was an opportunity to rest between blocks. All objects were presented at least once during practice, and Patient SA was instructed to tell the experimenter if she had difficulty recognising any of the objects (she did not report any difficulty). There were an equal number of trials containing stimuli of each category (kitchen or toolbox), and an equal number of congruent and incongruent trials with targets of each category (kitchen or toolbox) in each block. Order of presentation was shuffled randomly and independently for each block, and which image of the target category was presented was determined randomly and independently on a trial-by-trial basis.

### 3.2 Apparatus

Stimuli were displayed on a 21 inch CRT monitor (1024 × 768) which the participant viewed binocularly from a distance of 60 cm. Stimulus timing and presentation was locked to the screen refresh rate of 100 Hz. Stimuli were presented using a PC running Presentation software (version 13.1; http://www.neurobs.com).

Responses were measured using two specially designed devices, constructed from a rolled aneroid sphygmomanometer cuff (Boso-clinicus I, ref: 030-0-111), inflated to 20 mmHg, connected to a pressure transducer. One device was held in each hand, and the participant was instructed to make their responses by making a short, sharp squeeze of the rolled cuff and then release their grip. Grip force was converted to voltage which was digitised and stored using a LabJack U3 HV data acquisition device with DAQFactory software. Data were sampled at 1000 Hz. The participant was encouraged to respond as quickly as possible while maintaining a high level of accuracy, but no response feedback was given during the experiment.

### 3.3 Data analysis

Continuous force recordings were locked to stimulus onset and epoched into periods of 2500 msec, beginning 500 msec before target onset. Data were smoothed using a simple 5-point moving average to reduce high-frequency noise. The resulting waveforms were baseline corrected on a trial-by-trial basis according to the average baseline activity for each response device during the 200 msec pre-stimulus period on each trial.

A response (either correct or incorrect) was said to have occurred in a trial if at any point after the target stimulus onset until the end of the trial, two criteria were satisfied: (i) the force measured was greater than 3 SDs from the mean force measured during the pre-stimulus baseline period and that was followed by at least 18/20 points that also reached this threshold; and (ii) there was an increase in response by at least .01 V over the following 100 points or less. Response onset time (RT) was defined as the first point that satisfied these criteria. Peak response was determined as the maximum amplitude of the response made in a trial that was surrounded by points on either side with the same or lower amplitude.

Outliers were defined as any responses greater than three standard deviations (SDs) away from the mean response time for that hand, in that condition (congruent or incongruent) in that testing session. Remaining correct response times were entered into a 2 (hand) × 2 (congruency) × 2 (session) factorial analysis of variance (ANOVA). There was no significant effect of session (morning or afternoon) on RTs, and the effect of session did not interact with any of the other factors (all *F*'s < 1). Therefore, subsequent analyses collapse across session.

## 4 Results and discussion

**Fig. 3:**
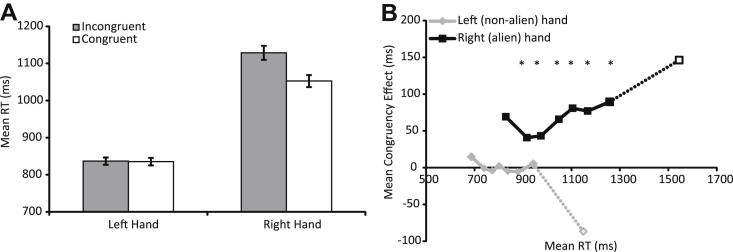
**Results from affordance task**. (A) Mean RTs (trimmed at +/− 3 SDs from the mean for each hand in each condition) for responses made with each hand to target stimuli that were incongruent (grey bars) or congruent (white bars) with the response afforded by the object. Error bars denote +/− 1 SEM. (B) Mean octile congruency effects as a function of mean RT for that octile, calculated separately for the left (grey line) and right (black line) hands. Data from the longest and most variable RT bin are included for completeness, and are shown here as dotted lines and open symbols. *Denotes Bonferroni-corrected *t*-test *p* < .05.

The key motivation in conducting Experiment 1 was to examine whether responses with Patient SA's alien hand were more susceptible to priming by object affordances relative to responses with her non-alien hand. Her responses were generally slower than those reported for healthy adults on this task (see McBride et al., 2012b). Moreover, SA's left (non-alien) hand responses were significantly faster than right (alien) hand responses [see Fig. 3; left mean = 836 msec *vs* right = 1090 msec, *F*(1, 497) = 307.47, *p* < .001]. Furthermore, stimuli which afforded a congruent response produced faster reactions than stimuli which afforded an incongruent response [incongruent mean = 983 msec; congruent mean = 944 msec; *F*(1, 497) = 7.13, *p* = .008]. Importantly, the congruency effect was much larger for the alien than for the non-alien hand [significant congruency × hand interaction: *F*(1, 497) = 6.62, *p* = .010]. This interaction is shown in Fig. 3A.

The congruency effect shown in the alien hand (76 msec) was several times larger than we have found using identical apparatus in healthy young controls (mean of median RTs = 16 msec, see McBride et al., 2012b). We also have as yet unpublished data on this task from elderly healthy controls (*N* = 26; aged 54–75 years; mean age = 64 years; one participant, who showed an average affordance effect of −111 msec, was removed as an outlier). As for Patient SA, we calculated each elderly control's mean RT for each condition, after removing outliers (following the same criteria for outlier selection as for Patient SA, we removed any RT that was greater than 3 SDs from each participants' mean RT for that hand for that condition). The elderly healthy controls had faster overall RTs (mean = 609 msec) and showed a smaller congruency effect [mean = 14 msec; congruency effect was reliable in elderly controls: *t*(24) = 3.15, *p* = .004] than for Patient SA's alien hand.^1^ To directly compare the performance of Patient SA's alien hand to that of healthy elderly controls, we converted the overall mean RT and affordance effect for the alien hand to *z*-scores, calculated according to the elderly controls' sample means and SDs. The *z*-scores for the affordance effect and overall RT shown for Patient SA's alien hand were 2.82 and 4.24, respectively. As these are both beyond the 95% limits (two-tailed) of the controls' distributions (95% limits are indicated by a *z*-score of 1.96), it is unlikely that Patient SA's effects are simply an extreme case in the normal elderly distribution, and that these effects are due to age.^2^

To investigate how often differences like those exhibited by SA's alien limb exist in healthy controls, we analysed the individual affordance effects for left and right hands in the young healthy controls previously reported by McBride et al. (2012a), plus the previously unpublished data from elderly healthy controls, mentioned above. None of these healthy adults showed the same pattern of effects shown by SA, with a significant interaction between the effects of hand and congruency, and a significant asymmetry in overall RT.

However, overall RTs in SA's alien hand were longer than those recorded in the non-alien hand, as well as those reported in young and elderly controls. Therefore, we performed further analyses to investigate the possibility that the difference in congruency effect across Patient SA's hands was simply attributable to the difference in baseline RT. We re-plotted the congruency effect as a function of RT in a *delta plot* (see van den Wildenberg et al., 2010, for a review of this technique and its advantages). For each hand separately, untrimmed (including those trials considered “outliers” for the ANOVA analysis) correct RTs were divided according to trial congruency (congruent or incongruent), rank-ordered, and then divided into eight bins of equal size. On two trials, no correct response was detected. Data for these trials were replaced with the mean correct RT for that hand and condition (this is a means to keep the total number of trials the same in each condition and dividable by 8, to avoid problems associated with unequal bin sizes). The mean RT in each bin for each condition was then calculated and the difference between incongruent and congruent trials is plotted against the mean RT for that bin (see Fig. 3B), giving the size of the congruency effect for that part of the RT distribution. Untrimmed RTs such as these typically have long “tails” produced from a number of slow outlier responses (see also Maylor et al., 2011). The increased variability and reduced reliability of long RTs mean that it is difficult to draw meaningful conclusions from the last bin, but it is included in the figure for completeness (dotted lines).

If the difference in congruency effects across the two hands was simply in line with differences in baseline RT, then we might expect similar congruency effects in those parts of the RT distribution which overlap across the two hands (i.e., for responses which onset between approximately 800–1000 msec after the stimulus appeared). However, there is clear separation between the congruency effects shown by the left and right hands in this part of the RT distribution, so it seems unlikely that the interaction between the effects of hand and congruency is being driven by differences in baseline RT. Thus, Patient SA shows a significantly larger affordance congruency effect when making responses with her alien (right) hand, compared to her non-alien (left) hand, suggesting that an object's affordance had an exaggerated effect on her alien limb compared to the unaffected hand.

The stimulus-response mappings Patient SA used in Experiment 1 were held constant over the course of the experiment. This was to prevent any possible difficulties Patient SA might have experienced with task-switching if we had changed the stimulus-response mapping part-way through the experiment (see Alvarez and Emory, 2006, for discussion). To examine whether there is any difference in the affordance effects normally produced by different stimulus types, we analysed affordance effects to these same stimuli from young (previously reported in McBride et al., 2012b) and elderly (previously unpublished) healthy control participants, where stimulus-response mapping was counterbalanced across participants. Young and elderly healthy controls showed comparable affordance effects for kitchen and toolbox stimuli [young controls' mean affordance effect for kitchen stimuli = 18 msec; for toolbox stimuli = 15 msec; no reliable difference of stimulus type on affordance effect: *t*(24) = .55, *p* = .59; elderly controls' mean affordance effect for kitchen stimuli = 12 msec; for toolbox stimuli = 16 msec; *t*(24) = .570, *p* = .574]. Therefore, there is no indication that there is any reliable difference in the affordances elicited by different stimulus types.

As noted in the methods, the particular object presented was determined randomly and independently for each trial (while the number of trials in each condition was held constant). Therefore, perhaps the very large affordance effect shown in Patient SA's right (alien) hand is due to a subset of toolbox stimuli which by chance appeared more (or less) often than the others. To investigate this possibility, we calculated how often each particular toolbox object was presented. Four out of the five objects appeared 54 or 55 times each, and one item (the chisel) was presented 39 times. Reassuringly, there was no reliable interaction between the affordance effect and the particular toolbox exemplar presented [congruency × object interaction: *F*(4, 239) = 1.20, *p* = .31]. Furthermore, we repeated the analysis of correct RTs after removing those trials which contained the relatively infrequent exemplar (the chisel). The affordance effect shown for the remaining toolbox items remains very large and statistically significant (incongruent mean = 1122 msec; congruent mean = 1064 msec; congruency effect = 58 msec, *p* = .03).

Errors were very infrequent (an above-threshold response was made by the erroneous hand on only 10/512 trials – approximately 2% of all trials). This error rate is similar to that which we observed in young (approximately 5%), and elderly (approximately 3%) healthy controls. Of these errors made by Patient SA, 8/10 were made by the right (alien) hand when the task required a response with the left hand. Six errors were detected by the alien limb in response to affordance incongruent trials (in other words, when the object presented required a left hand response, but was oriented such that it afforded a right-hand response), and 2 errors in response to affordance congruent trials. Errors were not confined to one particular stimulus, and instead were spread across 7 different exemplars. As errors were so infrequent, they were not analysed any further.

## 5 Experiment 2|Masked priming task

In Experiment 2, we used a backwards masked priming task (adapted from Sumner et al., 2007) to investigate automatic inhibition of responses that had been automatically primed in the alien and non-alien hands.

In order to be sure of producing automatic priming and inhibition of responses, it was necessary to change the interval between masked-prime and target. There are several methods reported in the literature to achieve this. One possibility would be to present the target stimulus once the mask had offset, and change the duration of the mask. However, shorter masks would be expected to mask the prime stimulus less well, which could have strong effects on the priming of responses. Alternatively, some researchers have used meta-contrast masking – that is, to use a stimulus which masks the prime by surrounding it. However, such masks are problematic because masks can act as prime stimuli in their own right – as masks of this type typically contain elements of both possible primes, any NCE obtained using such a mask may not be produced by response inhibition, but by mask-induced priming of the response opposite that evoked by the prime (see “object updating” e.g., Lleras and Enns, 2004; Sumner, 2008). As we were interested in the effects of automatic response inhibition, we sought to avoid this possibility.

Thus, we followed a well-established, standard method reported previously in the literature which is known to reliably produce PCEs and NCEs and which keeps the durations of each stimulus (prime, mask, and target) constant. We presented masks and primes simultaneously in short stimulus onset asynchrony (SOA) conditions, and introduced a blank screen between mask and target in long SOA conditions (see e.g., Boy et al., 2010a; 2010b; Boy and Sumner, 2010; Schlaghecken et al., 2006, 2003; Schlaghecken and Eimer, 2002; Schlaghecken and Maylor, 2005). It is possible that differences in the short- and long-SOA trial sequence may affect global RTs – for example the offset of the mask in the long SOA condition may serve as a warning signal that the target is about to appear and thus speed responses in the long SOA condition. However, as such effects are expected to have a global influence on RTs, and not affect one condition (compatible or incompatible) or hand (alien or non-alien) more than the other, they should not be able to account for any differences in compatibility effect shown in the different hands.

### 5.1 Stimuli and task

Each trial began with presentation of a white fixation cross on a mid-grey background. This cross subtended 1 degree × 1 degree of visual angle, and was presented in the centre of the screen for 500 msec. Following a blank interval of 200 msec, the prime appeared in the centre of the screen and remained for 50 msec (see below for how this duration was determined). The prime was then replaced with the mask which remained on the screen for 100 msec. Two mask-target SOAs were used in this experiment; 20 msec (short SOA, which was expected to produce a PCE) and 150 msec (long SOA, which was expected to produce an NCE). SOA conditions were presented in alternating blocks, starting with a long SOA block. Patient SA completed 8 blocks (4 of each SOA condition) of 84 trials each, making a total of 672 trials.

**Fig. 4:**
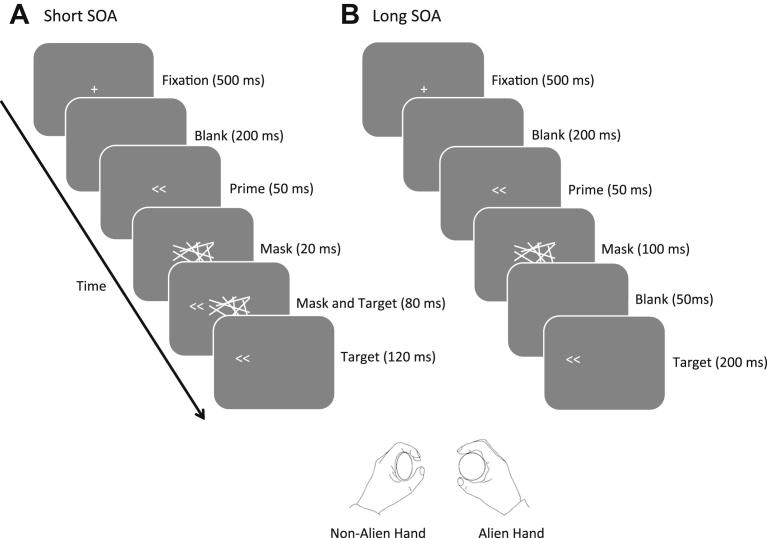
**Stimulus sequence and relevant timings in the masked-prime task**. In this example, as prime and target are associated with the same response, both trials shown are compatible trials. The patient made speeded squeeze responses according to the direction of the target presented on each trial (all trial types were equiprobable). (A) Shows the trial sequence for short SOA blocks, and (B) shows the trial sequence for long SOA blocks. The target arrowheads appeared 5 degrees of visual angle either to the left, right, or above the centre of the screen. All trial types were equiprobable. A left hand squeeze response to left-pointing target, prime compatible trials are shown here. Note that the mask and target were each presented for the same total time in both short and long SOA conditions.

A schematic of the stimuli and timings for this task can be seen in Fig. 4. Note that the total presentation time of each stimulus (prime, mask, target) was the same in both SOA conditions.

The target stimulus appeared after the mask had onset, and was either a left-, or right-pointing double arrowhead (so that it was either compatible or incompatible with the prime stimulus). The target appeared in one of three possible locations, centred 5 degrees of visual angle to the left, to the right, or above the centre of the screen. The participant was instructed to ignore the target's position, and to respond to the direction of this arrowhead by squeezing with either the left hand (for left-pointing targets) or the right hand (for right-pointing targets) as quickly and accurately as possible. In each block of trials there were an equal number of trials with each target type (left-, and right-pointing) in each possible position (left-, right-, above-centre), with each prime type (compatible and incompatible), presented in randomly shuffled order determined independently for each block. The target stimulus remained on the screen for 200 msec. There was a blank intertrial interval (ITI) of 2500 msec before the next trial began with the fixation cross. Data recording and analysis procedures were the same as those used in the affordance experiment.

Left- and right-pointing double arrowheads (e.g., “<<” and “>>”) served as primes and targets. The lines making up these stimuli were each 1 degree of visual angle long, and the lines in each arrowhead had an angular separation of 60° (30° above and below the horizontal). Masks were constructed of 30 pseudo-randomly orientated lines arranged into a 6 × 5 grid centred over the centre of the screen. To prevent any perceptual interactions between prime and mask modulating priming effects (see “object updating” accounts of the NCE e.g., Lleras and Enns, 2004) lines in the mask avoided any orientation within 5 degrees of the lines making up the prime and target. The lines in the mask were between 1.5 and 3 degrees of visual angle long. Line length and orientation were determined randomly within these limits and independently for each line in the mask. Thus, the mask was between 3.5 × 3.5–5.5 × 5.5 degrees of visual angle, centred on the centre of the screen. A new mask was constructed on each trial to prevent perceptual learning of the mask which could in turn lead to increased prime identification (e.g., Schlaghecken et al., 2008). Such masks have been shown not to invoke NCEs by object updating (Sumner, 2008) or by perceptual interactions (Boy and Sumner, 2010), and thus any NCEs observed can be attributed to motor inhibition.

### 5.2 Procedure to determine threshold for prime perception

Prior to the main experiment, the duration of the prime was set to below the threshold for conscious perception (50 msec duration) using a psychophysical staircase procedure. Here, on each trial a prime and mask were presented with no target, and the participant was instructed to make a 2-alternative forced-choice button-press according to the direction of the prime stimulus. The participant was instructed to make their best guess if they were unsure of prime direction, to concentrate on being accurate, and that speed was unimportant for this part of the task. The prime duration began at 120 msec, and then was varied according to a fixed-step, 1-up/2-down procedure: After two consecutive correct responses to primes presented at the same duration, prime duration was reduced by 10 msec on the next trial; after an incorrect response it was increased by 10 msec, within a range of 10–200 msec. This staircase procedure terminated after 10 “reversals”. The fastest prime duration was 60 msec (which was presented twice, and the prime was incorrectly identified on the second presentation), and the mean prime duration at the reversals was 84 msec. Thus, for the remainder of the experiment the prime duration was set to 50 msec, which was the faster than the fastest prime duration measured during the staircase (and was not reliably identified), and faster than the average duration of the reversals.

### 5.3 Prime-locked analysis of RTs

We followed the method described in Schlaghecken et al. (2011) to analyse RTs in a masked priming task relative to *prime* onset rather than to target onset. Schlaghecken and colleagues noted that the increased trial-to-trial variability in older adults' RTs may obscure the priming effects that would be revealed by traditional analyses which separate target-locked RTs according to prime-target compatibility and mask-target SOA on each trial. In fact, Schlaghecken et al. (2011) showed that calculating RTs relative to prime onset could reveal a reliable NCE in older participants' RTs when these were not shown by traditional analyses.

This method of analysis is essentially like the delta plot method used to analyse the data in Experiment 1. Trials in which no correct response was detected were replaced with the mean correct response time for that hand, condition, and mask-target SOA (this is a means to keep the total number of trials the same in each condition and dividable by 8, to avoid problems associated with unequal bin sizes). Then, response times were re-calculated relative to the *prime* onset (we added the prime-target SOA for each trial to the RT for that trial), and rank-ordered for each hand (left or right) for each condition (incompatible or compatible) *across* SOA conditions. This meant that there was some re-shuffling of responses across SOA conditions (because a slow response on a short SOA trial may have a longer prime-locked response time than a fast response on a long SOA trial). These prime-locked response times were divided into 8 bins of equal size. The average compatibility effect (average incompatible RT − average compatible RT) for each bin for each hand was calculated, and plotted relative to the mean RT for that bin and hand. Lastly, the statistical significance of the compatibility effect in each bin was determined by conducting a Bonferroni-corrected unpaired *t*-test on the response times in each bin.

## 6 Results and discussion

**Fig. 5:**
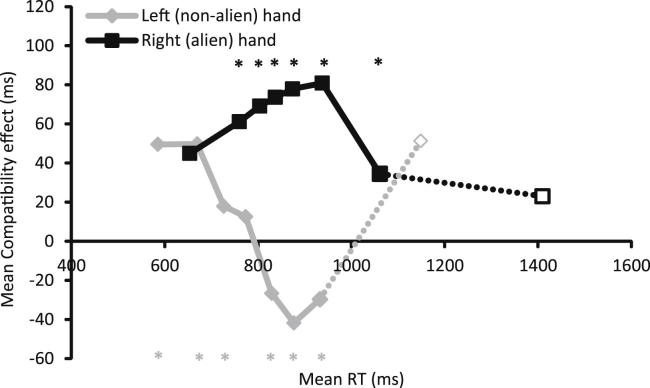
**Results from masked priming task**. Mean octile compatibility effects as a function of mean RT (relative to prime onset) for that octile, calculated separately for the left (grey line) and right (black line) hands following the procedure described in Schlaghecken et al. (2011). Data from the longest and most variable RT bin are included for completeness, and are shown here as dotted lines and open symbols. The left (non-alien) hand shows a pattern of compatibility effects which is similar to that reported elsewhere in healthy controls (PCE followed by NCE). The right (alien) hand shows a strong PCE, and there is no evidence that this turns negative as the prime-response interval increases. *Denotes Bonferroni-corrected *t*-test *p* < .05.

The results for the masked-prime experiment are shown in Fig. 5. The unaffected left hand showed the pattern of RT effects that would be expected from healthy individuals in a masked priming task (e.g., Schlaghecken and Eimer, 2002). For fast responses (which occurred most quickly after the prime was presented), RTs were faster for compatible trials relative to incompatible trials (a PCE). For responses that occurred later, responses were faster on incompatible trials relative to compatible trials (a NCE). There is some evidence that the priming effect may have returned to positive again at the tail end of the distribution in bin 8, which is also consistent with previous studies (see e.g., Sumner and Brandwood, 2008), but this effect may have been skewed by outliers in the tail end of the distribution, and did not reach statistical significance (Bonferroni-corrected *p* > .1).

A very different pattern emerged in the RTs for the responses made with the alien hand. Here, responses were consistently faster for compatible trials relative to incompatible trials (a PCE), and there was no evidence of this effect turning negative (NCE), even at later points in the distribution. While there is a small difference in the overall RTs for the left and the right hands, responses made with the left hand showed a significant NCE by around 850 msec after the prime had onset, whereas right-hand responses still showed a significant PCE at 1050 msec. Thus, these distributions suggest that this difference in compatibility effect is not likely to be due to the slightly longer right than left hand responses.

Furthermore, we suggest that it is unlikely that the inhibition has simply been delayed in right-hand responses. Maylor et al. (2011) reported reliable NCEs for elderly participants for responses which occurred by 500 msec after prime onset, whereas Patient SA here showed a priming effect that was still positive for responses which were recorded around 1050 msec after the prime had onset.

Schlaghecken et al. (2012) have recently suggested that prime-locked distributional analyses like those performed here can produce ‘significant’ effects in some latency bins by chance. Here we do not rely on searching for significant bins, but rather compare the whole pattern between the alien and non-alien hands. Nevertheless, we have also tested the possibility that the pattern shown by the alien hand could arise by chance from a ‘healthy’ distribution of data. We pooled the prime-locked RT data from the non-alien hand across compatible and incompatible conditions and randomly re-labelled trials as incompatible and compatible. We then re-ran the distributional analyses described here. After repeating this process 100 times, none of the 100 randomly re-sampled data sets showed the same reliable PCE in 6/8 RT bins as shown by the alien hand (and only 3 out of 100 showed a reliable PCE in any of 5/8 bins). Thus, we suggest that it is very unlikely that responses from Patient SA's alien hand actually belong to the same distribution as that of her non-alien hand, and only showed a consistently significant PCE due to chance.

Thus, there is no evidence of automatic motor inhibition of primed responses, indexed by the NCE, for responses made with the alien hand. It is unlikely that this disrupted inhibition is merely due to age or non-specific effects of disease, because reliable inhibition is shown for responses made with the left (non-alien) hand.

### 6.1 Spatial congruency effects

The design of the masked priming experiment required the target stimulus to be presented in a different location to the prime and mask (to avoid spatial and temporal overlapping of stimuli in the short SOA condition). Thus, on each trial the target was presented to the left, to the right, or above central fixation. This spatial aspect of the target stimulus might have affected performance in a manner similar to the Simon effect (e.g., Lu and Proctor, 1995, for a review) and the spatial Stroop effect (e.g., Banich et al., 2000). Thus, the design of this experiment provides an opportunity to investigate any effect of spatial congruency in Patient SA.

**Fig. 6:**
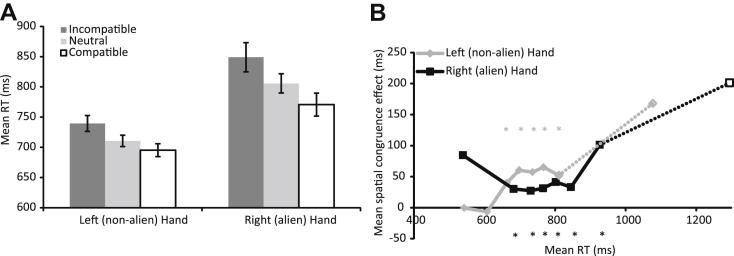
**Simon effect analysis**. (A) Mean RTs (trimmed at +/− 3 SDs from the mean for each hand in each condition) for trials in the masked priming task that were spatially incongruent, neutral, and congruent. Responses made with both hands show the expected spatial congruency effects, which were not significantly different for the left and right hands (see text for further details). Error bars denote +/− 1 SEM. (B) Mean octile spatial congruency effects as a function of the mean RT for that octile. Calculated by subtracting congruent RTs from incongruent RTs for each octile, for each hand separately. As above, data from the longest and most variable RT bin are included for completeness, and are shown here as dotted lines and open symbols. *Denotes Bonferroni-corrected *t*-test *p* < .05.

After removal of any response which occurred +/− 3 SDs away from the mean of that condition, we calculated mean RTs for each hand for spatially incongruent, neutral, and congruent trials. These mean RTs are shown in Fig. 6A. The expected location congruency effects were observed: responses were fastest when the target appeared in a location that was congruent with the required response, and slowest when the target appeared in a location that was congruent with the response opposite that required to the target {incongruent condition; [*F*(2, 627) = 7.37, *p* = .001]}. Also, as expected, responses made with the left (non-alien) hand were significantly faster than responses made with the right (alien) hand [*F*(1, 627) = 51.12, *p* < .001]. Importantly, the interaction between the effects of hand and congruency did not approach statistical significance [*F*(2, 627) < 1].

As noted above, a delta plot can be a more sensitive way of examining RT effects than comparing average RTs. Therefore, we have plotted the spatial congruency effect (incongruent RT − congruent RT) over 8 RT bins (see Fig. 6B) according to the procedures described above. The pattern of spatial congruency effects was similar for both hands, and the effect did not reach significance at the beginning or end of the distribution for either hand.^3^

In summary, there is no evidence that the spatial congruency effects on RT were different for the alien and non-alien hand.

### 6.2 Errors

Error responses were detected in 9.8% of all trials in the Masked Priming task. Table 2 shows how many trials of each type (divided by prime-target SOA, prime-target compatibility, and location-target congruence) contained an erroneous response (out of a maximum of 28 trials in each cell). Note that trial types are divided according to the correct response, so for example an error occurring on a prime incompatible trial means that the prime was incompatible with the correct response required to the target (and so primed a response in the incorrect hand).

As shown in Table 2, most errors were observed in the right (alien) hand in response to a target requiring a left hand response (62/66 errors were of this type). These errors were more frequent when the target was in the incongruent (i.e., rightward) location – suggesting that the patient might have been responding to the location of the target rather than to its identity. The pattern of errors suggests that there may have been a hint of an interaction between the effects of hand and spatial congruency on error rates. However, as there were so few errors detected in the left (non-alien) hand, we cannot meaningfully compare erroneous left- and right-hand responses in different conditions.

## 7 General discussion

Continuous force responses from both hands of a single patient with AHS due to CBS were measured while she completed two experimental tasks designed to investigate automatic action priming and control. The results presented here show two potentially theoretically important findings. First, there was an exaggerated affordance congruency effect when the patient made responses with her alien (right) hand compared to her unaffected (left) hand. Second, we found no evidence of automatic inhibition of primed responses in her alien hand, despite a normal inhibitory effect in the non-alien hand. However, in contrast, there was no reliable difference in the Simon/spatial-Stroop congruency effects on RTs for responses made with the two hands.

In healthy observers, there is good evidence that perceptual processing of even an image of a graspable object automatically primes the action that has been associated with that object (see e.g., Grèzes and Decety, 2002; McBride et al., 2012b; Tucker and Ellis, 1998). Our finding that this effect is exaggerated for responses made by an alien hand relative to the unaffected limb supports the suggestion that patients with alien hand are particularly susceptible to overlearned stimulus-response associations (affordances), even when they conflict with current task demands (see also Riddoch et al., 1998).

The SMA in the medial frontal lobe may play an important role in mediating automatically evoked action priming by objects in the environment. Significant activity in the SMA has been demonstrated when healthy observers simply view objects without initiating actions (e.g., Grèzes and Decety, 2002), and damage to this region is associated with CBS (e.g., Garraux et al., 2000) and AHS (e.g., Marchetti and Della Sala, 1998). Activity in the SMA has also been associated with automatic inhibition of automatically primed responses (e.g., Boy et al., 2010a). There was no sign of such automatic inhibition of responses in Patient SA's alien hand, even though this process seemed to be intact for their non-alien hand.

AHS has been characterised – at least in part – as a failure to execute endogenous or volitional control over actions afforded by the environment (e.g., Biran et al., 2006; Giovannetti et al., 2005). However, in the masked priming task used here, the patient was not instructed to inhibit responses that were evoked by the prime stimulus. Indeed, the prime was presented subliminally, so it is reasonable to assume that Patient SA cannot have been aware of which direction the prime pointed in order to endogenously halt any motor activation it produced. Thus, the absent NCE in the masked priming task reported here might suggest that there is disruption to *automatic* and *unconscious* inhibition of primed actions in Patient SA's alien hand.

The NCE is thought to reflect a mechanism of automatic self-inhibition (see Boy et al., 2008). The motor inhibition indexed by the NCE does not transfer across effectors (e.g., Eimer et al., 2002; see also Sumner et al., 2007) and does not seem to act on individual muscle commands. Instead, it affects abstract response representations, most likely upstream of the primary motor cortex (Schlaghecken et al., 2009). Therefore, the findings presented here suggest that Patient SA can form stimulus–action associations, which can be partially activated by masked primes, and that unwanted right (alien) hand primed actions are not successfully inhibited.

Sumner and Husain (2008) have recently proposed that automatic inhibitory mechanisms may contribute to flexible, goal-driven behaviour by rapidly suppressing unwanted actions which have been automatically and exogenously activated by the environment. Such inhibition may create a level playing field on which all possible actions can compete for selection according to intentions. Indeed, if disrupted suppression of unwanted actions leaves AHS patients at the mercy of actions which have been afforded by their environment, this may go some way to account for many of the grasping behaviours shown in these patients. Of course, it is possible that the inhibitory mechanisms indexed by the NCE and action priming effects shown in object affordance are not related as we have suggested, and instead are independent. Future work in this area could better characterise any relationship between automatic inhibition and object affordance by correlating the size of object affordance effects and NCEs in a large group of alien hand patients.

There may also be disruption to endogenous (intention-driven) control of actions in AHS (as suggested by e.g., Biran et al., 2006; Giovannetti et al., 2005). Schaefer et al. (2010) recently examined the neural correlates of unwanted movements in AHS, and found that the right inferior frontal gyrus (rIFG) was activated only during alien movements. This brain region has been associated with endogenously-driven inhibitory control over motor responses which have already been programmed or partially executed in the stop signal task (e.g., Aron, 2007; Hampshire et al., 2010; Swann et al., 2009, 2012; Verbruggen et al., 2010). Thus, such rIFG activation might arguably reflect unsuccessful endogenous attempts to inhibit “alien” movements.

### 7.1 Additional considerations

Of course, the results reported here were gathered from a single case of CBS with AHS. As with all single case reports it is possible that the tested case is not qualitatively unusual relative to healthy controls, and instead represents an extreme result drawn from the normal distribution. To go some way to addressing this issue we have shown that the affordance effects shown by Patient SA's alien hand are beyond the 95% confidence limits of the distribution of effects shown by elderly healthy controls. Furthermore, no single healthy control (young or old) showed the same overall pattern of results as the patient (even with numerically smaller effects). Thus, it is unlikely that the affordance effect shown in Patient SA's alien hand represents an extreme case in the normal distribution. One could also address this issue by showing the same result in more cases of CBS with AHS. However, CBS is an extremely rare (as noted above, annual incidence rates have been estimated at around .02 per 100,000 individuals; Winter et al., 2010) and degenerative disease, and although we saw a further three patients with CBS, their motor symptoms were so severe that they could not carry out the tasks described here.

The neural mechanisms which give rise to AHS are not clear, and a range of phenomena (see Table 1, for possible examples) have been reported in patients with AHS. The single case we have presented here experienced grasping of objects placed within her reach, but not arm levitation, intermanual conflict, mirror movements, or self-choking (but it is possible that the very rare descriptions of choking are simply a very extreme form of the involuntary grasping we have observed). Therefore, while the data presented here suggest that disrupted automatic inhibition may contribute to involuntary grasping behaviour in AHS, it is not clear how far results from this single case can be generalised to different variants of AHS, and AHS produced by lesions in different brain areas (such as from medial frontal areas e.g., Bakheit et al., 2013; Garraux et al., 2000; Marchetti and Della Sala, 1998; and posterior parietal regions e.g., Coulthard et al., 2007).

Additionally, it is worth considering other possible explanations for the effects reported here. First, in Experiment 1, the location of the action-affording property of the objects presented (the handles) may be confounded with the visually most salient part of the stimulus. Thus, the effect which we have interpreted as “affordance” may instead reflect compatibility between the location of the most perceptually salient part of the image, and the location of the response (i.e., see Anderson et al., 2002). However, we directly investigated spatial congruency effects shown by Patient SA using data from the masked priming task, and showed that there was no significant difference in the spatial congruency effects shown in the time taken for the patient to respond using the left and right hands. Although it is not possible to comprehensively rule out any interaction of spatial congruency and hand in Patient SA, as it was not possible to statistically test the effects of spatial congruency on error rates with the left and right hands, if spatial congruency is to explain the RT results of the affordance experiment, there is no obvious reason why such an effect would be absent in the RTs of the priming experiment.

Second, responses made with Patient SA's alien hand were significantly slower than responses made with the non-alien hand, particularly in the object affordance task. Therefore, one could suggest that the different affordance effects reported for the alien and non-alien hands are simply proportional to the differences in baseline RTs between the two hands. As different congruency effects were shown for overlapping portions of the RT distributions for the left and right hands for Patient SA (see Figs. 3 and 5), we suggest this is unlikely.

However, as Patient SA reports difficulty using her right arm, she may have learned to avoid using it which may in turn have produced longer RTs for responses made by the alien hand relative to the non-alien hand independently from any effects of AHS. However, it is not clear how any such learned avoidance could produce the patterns of PCEs and NCEs shown in Experiment 2. In order for the NCE to be absent – perhaps due to motor processes becoming weaker when unused, or due to tonic inhibition of responses in the alien hand – we would also expect the PCE to be similarly absent or reduced, which was not the case. Alternatively, perhaps learned avoidance resulted in a general difficulty in using the alien hand, especially when the stimulus primes a response in the opposite hand. This could contribute to affordance effects reported in Experiment 1 and the PCE in Experiment 2, but would also have been expected to generalise to spatial congruency effects, which was not supported by our data. Nevertheless, the best way to test for learned avoidance behaviour in AHS would be to follow a patient longitudinally from before diagnosis to discover whether such effects emerge *after* the alien limb symptoms. While this was not possible with the patient reported in this paper because we did not assess her at the time of the very earliest symptoms, it may be a fruitful avenue for future research.

Third, one could argue that the absent NCE in the alien hand does not reflect absent automatic inhibition, and instead that the primed responses were so strongly activated that the (intact) inhibitory mechanisms were insufficient to prevent the primed response being executed. For this to explain the absent NCE in the alien hand, we would also have expected a larger PCE over the earliest RT bins compared to the non-alien hand (which was not the case here, see Fig. 5).

Fourth, one could suggest that differences in stimulus presentation between the short- and long-SOA conditions in the masked priming task could have affected responses. For example, perhaps the delay between the mask and target in the long SOA condition may have allowed for better attentional disengagement from the preceding mask relative to the short SOA condition. Such attentional disengagement would be expected to speed responses when SOAs were long. Similarly, perhaps crowding or flanking effects from the mask would have lengthened RTs to targets in the short SOA trials (where masks and targets were presented simultaneously) relative to the long SOA trials. Again, this would be expected to produce a global slowing of RT in the short SOA condition. However, both of these global effects on RT would not be expected to differentially affect compatible and incompatible trials, or left and right targets, so they cannot account for the observed effects reported here.

Finally, perhaps differences in affordance and masked priming effects across the hands in Patient SA occurred by chance, and are not related to her neurological condition. To investigate how often differences like those shown in SA exist in healthy controls, we analysed data from healthy people, including an elderly group, on our affordance task and found that none demonstrated the same pattern of results shown by Patient SA. This suggests the effects in SA are related to her AHS or CBS.

### 7.2 Conclusion

In summary, we provide evidence from a single case study that (1) motor responses made with an alien hand may be hyper-sensitively modulated by affordances, and (2) that there may be disruption of automatic and unconscious inhibition of unwanted actions in the alien hand. Such disruption may go some way to explain the involuntary grasping behaviour shown in some patients with AHS, even when such grasping actions conflict with their intentions.

## Figures and Tables

**Table 1:** **Patient details**. Details of the four patients with CBS. Patients FC, DH, and EF had motor deficits that were so severe that they were not able to perform simple motor tasks. The experiments reported here were conducted only with Patient SA. + Indicates that a symptom was present, – indicates that the symptom was not detected.

	Patient SA	Patient FC	Patient DH	Patient EF
Demographics				
Age (years)	72	70	61	59
Sex (M/F)	F	F	M	F
Manual symptoms				
Involuntary grasping	+ Right hand	+ Both hands	–	+ Left hand
Difficulty releasing	–	+ Both hands	–	–
Intermanual conflict	–	–	–	–
Arm levitation	–	+ Both hands	–	–
Mirror movements	–	–	–	–
Dystonia	–	+ Both hands	–	–
Dyspraxia	–	+ Both hands	+ Some in left hand	+
Tremor	–	–	–	–
Rigidity	+ Right arm	+ Both hands	+ Especially left arm	+
Impaired eye-movements				
Voluntary saccades				
Horizontal	Slowed to left	Impaired	–	–
Vertical	–	Impaired	Impaired	–
Reflexive saccades				
Horizontal	–	Impaired	–	Impaired
Vertical	–	Impaired	–	–
Other symptoms				
Telegraphic speech	+	+	+	+
Visual extinction	–	+	–	–
Tactile extinction	–	+	+	–

**Table 2:** **Masked-prime error data**. Number of trials of each type (out of a maximum of 28 in each cell) which contained a response with the incorrect hand (error). (A) Shows the number of errors made with the left (non-alien) hand; (B) Shows the number of errors made with the right (alien) hand.

	Short SOA		Long SOA	
Prime incompatible	Prime compatible	Prime incompatible	Prime compatible
(A) Errors made with the left (non-alien) hand				
Location incongruent	0	0	1	1
Location neutral	1	0	0	0
Location congruent	1	0	0	0
(B) Errors made with the right (alien) hand				
Location incongruent	6	2	9	11
Location neutral	1	1	9	6
Location congruent	4	2	5	6

## Footnotes

1 We have also examined the median affordance effects from untrimmed RTs. For Patient SA: median affordance effect non-alien hand = 7 msec; median affordance effect alien hand = 67 msec; for healthy elderly controls: mean of median affordance effect = 13 msec.

2 Recently, Crawford and Garthwaite (2012) have suggested an alternative method for testing whether an effect shown by a single case is likely to have been drawn from the control distribution by conducting a *t*-test following Crawford and Howell's (1998) method. For completeness, we have also conducted this test, which also indicates that the affordance effect [*t*(24) = 2.76, *p* < .01] and overall RT [*t*(24) = 4.16, *p* < .01] for Patient SA's alien hand are reliably different from those shown in elderly controls (tests are two-tailed).

3 Different spatial congruency effects have been shown to produce different patterns in RT delta plots (e.g., Pratte et al., 2010). Simon effects typically produce negatively-sloping delta plots (due to inhibition of automatically location-activated responses which develops over time, see van den Wildenberg et al., 2010, for a review). Spatial Stroop effects generally produce positive sloping delta plots. Delta plots for the left and right hands in Patient SA are both positive – suggesting that our task is more influenced by Stroop- rather than Simon-like effects.
